# A cohort study to evaluate persistence of hepatitis B immunogenicity after administration of hexavalent vaccines

**DOI:** 10.1186/1471-2334-8-100

**Published:** 2008-07-28

**Authors:** Cristina Giambi, Antonino Bella, Antonella Barale, Domenico Montù, Maria Marchisio, Maurizio Oddone, Salvatore Zito, Maria Rapicetta, Paola Chionne, Elisabetta Madonna, Marta L Ciofi degli Atti

**Affiliations:** 1 Communicable Disease Epidemiology Unit. National Centre for Epidemiology, Surveillance and Health Promotion. Istituto Superiore di Sanità, Viale Regina Elena 299, 00161 Rome, Italy; 2 University of Rome Tor Vergata, Viale Montpellier 1, 00133 Rome, Italy; 3 Regional Unit of Infectious Disease Epidemiology. Local Health Unit of Alessandria, Piedmont Region – Via Venezia 6, 15100 Alessandria, Italy; 4 Local Health Unit 17, Savigliano, Piedmont Region – Via Lancimano 9, 12045 Fossano (Cuneo), Italy; 5 Local Health Unit 19 Asti, Piedmont Region – Via Conte Verde 125, 14100 Asti, Italy; 6 Department of Infectious, Parasitic and Immune-Mediated Diseases. Istituto Superiore di Sanità, Viale Regina Elena 299, 00161 Rome, Italy

## Abstract

**Background:**

In 2001, two hexavalent vaccines were licensed in Italy (Hexavac^®^, Infanrix Hexa^®^), and since 2002 were extensively used for primary immunization in the first year of life (at 3, 5, 11/12 months of age). In 2005, the market authorization of Hexavac^® ^was precautionary suspended by EMEA, because of doubts on long-term protection against hepatitis B virus. The objectives of this study were to evaluate the persistence of antibodies to anti-HBs, in children in the third year of life, and to investigate the response to a booster dose of hepatitis B vaccine.

**Methods:**

Participant children were enrolled concomitantly with the offering of anti-polio booster dose, in the third year of life. Anti-HBs titers were determined on capillary blood samples. A booster dose of hepatitis B vaccine was administered to children with anti-HBs titers < 10 mIU/ml, with the monovalent precursor product of the previously received hexavalent vaccine. HBsAb titers were tested again one month after the booster.

**Results:**

Sera from 113 children previously vaccinated with Hexavac^®^, and from 124 vaccinated with Infanrix Hexa^® ^were tested for anti-HBs. Titers were ≥ 10 mIU/ml in 69% and 96% (p < 0,0001) respectively. The proportion of children with titers ≥ 100 mIU/ml did also significantly differ among groups (27% and 78%; p < 0,0001).

Post-booster, 93% of children achieved titers ≥ 10 mIU/ml, with no significant difference by vaccine group.

**Discussion:**

Fifteen months after third dose administration, a significant difference in anti-HBs titers was noted in the two vaccine groups considered. Monovalent hepatitis B vaccine administration in 3-year old children induced a proper booster response, confirming that immunologic memory persists in children with anti-HBs titers < 10 mIU/ml. However, long-term persistence of HBV protection after hexavalent vaccines administration should be further evaluated over time.

## Background

The hepatitis B vaccine is available since 1982, initially as a plasma-derived vaccine, and from 1984 onwards as a recombinant vaccine [1]. Since the late 1990s many combined vaccines which protect against hepatitis B virus (HBV) and other vaccine-preventable diseases have been licensed. In particular, in 2001 hexavalent products, which associate the hepatitis B component to the components against diphtheria, poliomyelitis, tetanus, pertussis and *Haemophilus influenzae *type b, were introduced in the European Union.

Currently available vaccines are safe and highly immunogenic. In fact, after the administration of three doses of vaccine, more than 95% of children develop antibodies to hepatitis B surface antigen (anti-HBs) ≥ 10 mIU/ml, considered, according to international standards, to be protective against this infection [1].

The duration of protection induced by plasma-derived and recombinant vaccines against HBV was investigated by several authors [2-12], and even if current data show a decline of antibody titers over time, there is evidence that immunological memory persists for at least 9–15 years after immunization [4,7,8,11-13].

On the basis of available data, the European Consensus Group on Hepatitis B Immunity in 1998 [14], the World Health Organization in 2002 [15], and the Viral Hepatitis Prevention Board in 2004 [13] concluded that there is no evidence to introduce a booster dose in universal hepatitis B immunization programs.

Recently, a correlation between the antibody titers observed one month after the administration of the third hepatitis B vaccine dose, and the long-term persistence of immunological memory has been shown [3,7,10,11]. Concerns have thus arisen about long-term protection of children who after vaccination present anti-HBs titers between 10 and 99 mIU/ml [10], and in 2005, the European Medicines Agency (EMEA) suspended, as a precautionary measure, the marketing authorisation for the hexavalent vaccine Hexavac^® ^(Sanofi Pasteur MSD) [16]. In fact, it was shown that although more than 95% of children vaccinated with Hexavac^® ^developed anti-HBs titers ≥ 10 mIU/ml one month after the third dose, 5–20% of vaccinees had antibody levels between 10 and 99 mIU/ml.

In Italy, Hexavac^® ^was licensed in 2001, along with another hexavalent vaccine (Infanrix Hexa^®^, SmithKline Beecham). These two vaccines were extensively used for primary immunization in the first year of life since August 2002, when the infancy poliomyelitis vaccination schedule was changed, shifting from a sequential scheme with two doses of live attenuated vaccine (OPV), plus one dose of inactivated vaccine (IPV), to three doses of IPV. According to this new schedule, infants are vaccinated with three doses of vaccines against diphtheria, tetanus, HBV, pertussis, polio, and *Haemophilus influenzae *type b, at 3, 5, 11/12 months of age. In each of the 21 Italian Regions, Local Health Units are responsible to purchase the vaccines, and they can choose any product available on the market.

The objective of this study was to evaluate the persistence of the immune response against hepatitis B virus in children vaccinated in the first year of life with a 3-dose course of available hexavalent vaccines. Children families were invited to participate when they were actively offered the polio vaccine booster dose, which, according to Italian schedule, should be administered in the third year of life.

## Methods

### Study population

This cohort, prospective study was conducted in two Local Health Units in Piedmont Region, Northern Italy, from May 2005 to June 2007.

The two Local Health Units were selected because they presented a similar organization of vaccination services, but they used different hexavalent vaccines (Hexavac^® ^and Infanrix Hexa^®^).

The planned sample size was 100 children previously vaccinated with Hexavac^® ^at 3, 5, 12 months of age, and 100 children who received Infanrix Hexa^® ^with the same schedule.

A total sample size of 200 children (100 for group) would allow for a 92% statistical power to detect a difference of 20% between groups (beta and alfa errors equal to 0.08 and 0.05, respectively).

The inclusion criteria were as follows: a) age between 2 and 3 years; b) vaccination by one year of life (12 ± 2 months of age) with three doses of the same hexavalent product (Hexavac^® ^or Infanrix Hexa^®^); c) interval from the last hexavalent vaccine dose ≥ 12 months.

Children were excluded if they were affected by a chronic illness, or by a congenital or acquired immune response disorder.

The study was approved by the ethical committees of the Local Health Unit in charge of infection disease control in Piedmont (Alessandria), and of the Italian Institute of Health (Istituto Superiore di Sanità, Rome). A written, informed consent was obtained from both parents of each participant child.

### Evaluation of immunogenicity and response to a booster dose

A capillary blood sample was collected at enrollment. Serum specimens were stored at -20°C, until testing for anti-HBs antibodies.

Serological testing was carried out at Istituto Superiore di Sanità. All the serum specimens were tested for quantitative anti-HBs antibodies using a commercial test kit based on the immunoenzimatic method MEIA (AxSYM AUSAB; Abbott Laboratories, Abbott Park, IL USA). The test was used according to the manufacturer's instructions.

Children with anti-HBs titers < 10 mIU/ml were offered a booster dose with a monovalent hepatitis B vaccine, using the vaccine produced by the same manufacturer of the previously administered hexavalent product, i.e.: HBVaxPro 5^® ^(Sanofi Pasteur MSD) for children vaccinated with Hexavac^®^, and Engerix-B 10^® ^(SmithKline Beecham) for children vaccinated with Infanrix Hexa^®^.

A second capillary blood sample was obtained one month after booster dose administration (30 ± 2 days). The presence of an anamnestic response was defined as a fourfold or greater rise in anti-HBs titers between pre- and post-booster administration, providing that a titer ≥ 10 mIU/ml was reached after the booster.

After booster administration, parents of participant children were also asked to fill-in a 8-day diary, including information on body temperature and onset of the following signs and symptoms: 1) local reactions (redness, swelling, pain and itching in the injection site); 2) systemic reactions (sleepiness, irritability, lack of appetite and vomiting).

### Statistical analysis

For each type of vaccine, we estimated the proportion of participants with anti-HBs titers < 10, 10–99 and ≥ 100 mIU/ml, and the geometric means titers (GMTs) with 95% confidence intervals (95%CI), before and after the administration of the booster dose.

For the calculation of GMTs, all the sera with anti-HBs titers ≥ 0.1 were included in the analysis and the highest titer was fixed to 2000 mIU/ml.

Differences in frequency between groups were compared with the Chi-square test or Fisher's exact test; t-test or Mann-Whitney nonparametric test were used to compare continuous variables.

Relative Risks (RR) with 95% confidence intervals were calculated. A Poisson multiple regression model with robust error variance was used to evaluate the role of covariates.. Variables which differed by study group at the univariate analysis (p < 0.10) were included into the multivariate model and retained in the final model according to a log likelihood ratio test for goodness-of-fit.

Statistical analyses were conducted by using STATA 8.2 (Stata Corporation, College Station, Texas, USA).

## Results

### Characteristics of participants

A total of 242 participants were enrolled: 113 previously vaccinated with Hexavac^® ^and 129 with Infanrix Hexa^®^.

**Figure 1:**
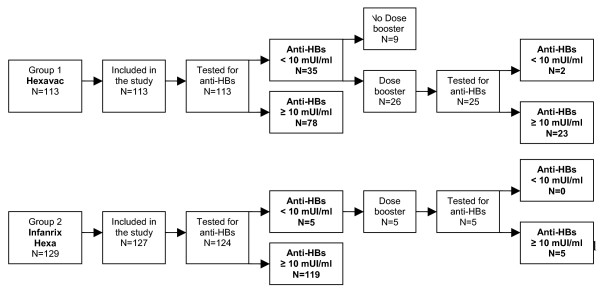
Description of study population.

Two participants were excluded from the analysis because they did not meet the inclusion criteria (i.e., one child received the third hepatitis B vaccine dose at 15 months of age; one child received the third dose < one year of enrolment). Additional three samples were not tested because serum was not sufficient (Figure 1 – Description of study population).

A total of 237 serum samples were thus analysed: 113 collected from children vaccinated with Hexavac^®^, and 124 from children vaccinated with Infanrix Hexa^®^.

The distribution by gender, gestational age, and birth weight were similar among vaccine groups. By contrast, mean age, mean interval from third dose, and proportion of children vaccinated with measles-mumps-rubella vaccine (MMR) significantly differed by group (Table 1 – Characteristics of participants).

**Table 1:** Characteristics of participants

	**Group 1 Hexavac**^®^	**Group 2 Infanrix Hexa**^®^	**p-value**
Number	113	124	-
Females, No (%)	44 (38.9)	58 (46.8)	0.2240
Mean age in months (± SD)	26.9 (± 1.2)	26.5 (± 1.3)	0.0232
Mean time in months since the third dose (± SD)	15.8 (± 0.9)	15.3 (± 1.5)	0.0093
Mean birth weight in grams (± SD)	3194.6 (± 560.8)	3233.2 (± 435.3)	0.9116
Mean gestational age in weeks (± SD),	39.3 (± 1.8)	39.2 (± 1.5)	0.4420
Vaccinees with MPR (%)	104 (92.1)	124 (100.0)	0.0010

### Persistence of immunogenicity

Of the 113 children previously vaccinated with Hexavac^®^, 31% had an anti-HBs titer below the threshold considered protective, compared with 4% of children of the Infanrix Hexa^® ^group (p < 0.0001).

The multivariate analysis shows that the type of vaccine was the only determinant of antibody titer, after adjusting for age, time-interval from the third hexavalent vaccine dose, and previous MMR vaccination.

Among the children with a titer ≥ 10 mIU/ml, 60% had a titer between 10 and 99 mIU/ml in the Hexavac^® ^group, compared with 18% in the Infanrix Hexa^® ^group (p < 0.0001) (Table 2 – Pre-booster anti-HBs titer by vaccine group).

**Table 2:** Pre-booster anti-HBs titer by vaccine group.

**Anti-HBs titer (mIU/ml)**	**Group 1 (N = 113) Hexavac**^® ^**n (%)**	**Group 2 (N = 124) Infanrix Hexa**^® ^**n (%)**	**p-value**	**RR (95%CI)**
< 10	35 (31.0)	5 (4.0)	< 0.0001*	7.7 (3.1 – 18.9)
10 – 99	47 (41.6)	22 (17.8)	< 0.0001**	3.3 (2.1 – 4.9)
≥ 100	31 (27.4)	97 (78.2)	-	-

* < 10 vs ≥ 10; ** 10–99 vs ≥ 100

Antibody GMTs were also significantly lower in children vaccinated with Hexavac^®^, compared to Infanrix Hexa^® ^[27.6 (95%CI 18.8–40.4) vs 332.2 (95%CI 245.0–450.3) mIU/ml; p < 0.0001)].

### Response to booster dose

A booster dose of hepatitis B vaccine was administered to 31/40 children with anti-HBs titers < 10 mIU/ml (26 previously vaccinated with Hexavac^®^, and 5 with Infanrix Hexa^®^).

Of the remaining 9 children, one was lost at follow up, while the parents of the other 8 declined participation.

One month after the booster vaccination, a second capillary blood sample was obtained from 30 children (Figure 1 – Description of study population).

Two children of the Hexavac^® ^group had a post-booster anti-HBs titer < 10 mIU/ml; all the other children met the criteria for the anamnestic response. There was no statistically significant difference between the two vaccines, in the proportions of children with a post-booster titer < 10 mIU/ml, and between 10 and 99 mIU/ml (Table 3 – Post-booster anti-HBs titre by vaccine group).

**Table 3:** Post-booster anti-HBs titre by vaccine group.

**Anti-HBs titer (mIU/ml)**	**Group 1 (N = 25) Hexavac**^® ^**n (%)**	**Group 2 (N = 5) Infanrix Hexa**^® ^**n (%)**	**p-value**
< 10	2 (8.0)	0	1.0000*
10–99	4 (16.0)	2 (40.0)	0.2854**
≥ 100	19 (76.0)	3 (60.0)	-

* < 10 vs ≥ 10; ** 10–99 vs ≥ 100

The only two children who did not respond to the booster dose were previously vaccinated with Hexavac^®^, and prior to the booster had undetectable titers.

Post-booster GMTs did not differ significantly by vaccine group: 178.0 mIU/ml (95%CI: 87.5–362.3) in children vaccinated with Hexavac^® ^vs 124.0 mIU/ml (95%CI: 27.0–568.0) in vaccinated with Infanrix Hexa^® ^(p = 0.4192).

The diary for recording vaccine side reactions was returned by 30 participants; parents of 20 children (67%) recorded at least one event. Fever was described in 13 children (43%), being the most commonly reported event. In most cases (10/13), fever onset was within 3 days of vaccination; two of the three children who developed fever after the third day were diagnosed with pharingitis and influenza. Local reactions were described in 4 participants (13%).

## Discussion

In this study, persistence of HBV immunogenicity was evaluated in 3-year old children, who are routinely invited to vaccination services for polio booster administration. In 2005, these children represented the first cohorts of individuals who were immunised in infancy with three doses of hexavalent vaccines.

Our results show a significant difference between the two products, with lower antibody levels in children previously vaccinated with Hexavac^®^, compared to those vaccinated with Infanrix Hexa^®^. In fact, in the Infanrix Hexa^® ^group the proportion of children with anti-HBs titers < 10 mIU/ml is consistent with the expected proportion of vaccine primary nonresponders (about 5%) [1], while it is eightfold higher among the children vaccinated with Hexavac^® ^(31%).

One of the limitations of our study is that anti-HBs titers after primary immunization are not available, so it is not known if children with pre-booster titers < 10 mIU/ml were vaccine primary non-responders or if their titers declined over time.

Other studies involving children vaccinated with recombinant hepatitis B vaccines administered in the first year of life, found that 36–95% of vaccinees had titers < 10 mIU/ml, 5–15 years after the primary course [6,8,12,17]. Nevertheless, these data are not directly comparable to our results, because of two main reasons: 1) these studies were conducted using monovalent vaccines, and 2) specific antibody titers were evaluated many years after the completion of the primary series. By contrast, our data refer to combined vaccines, and have been obtained shortly after third dose administration (at a mean interval of 15 months). Therefore, the proportion of individuals with titers < 10 mIU/ml might increase on a longer evaluation period.

The higher proportion of children with anti-HBs titers between 10 and 99 mIU/ml observed in the Hexavac^® ^group compared to the Infanrix Hexa^® ^group also suggests a potential further decline of the immunogenicity of this vaccine over time.

There is no evidence that vaccinated children showing anti-HBs titers < 10 mIU/ml, measured after more than 30 days from the third dose, are susceptible to HBV infection [1]. In fact, this value represents a cut-off indicative of seroprotection when antibodies are tested approximately 30 days after the third dose administration.

When HBV immunogenicity is evaluated at a longer interval from primary vaccination, the presence of specific immune memory in individuals with anti-HBs titers < 10 mIU/ml can be inferred by testing the response to a booster dose.

However, there is no standard definition of anamnestic response to hepatitis B vaccine [4-6,8,10,12,18-20]. Also the appropriate interval between the booster dose and the immune response testing is not univocally defined [4-6,10,17,20], but no appreciable difference has been demonstrated in the anamnestic response evaluated 2 or 4 weeks after booster administration [20]. We thus adopted the more frequently used definition, that is a fourfold or greater increase in anti-HBs titer providing that a titer ≥ 10 mIU/ml is reached [4-6], as measured one month after booster dose.

According to this definition, 93% of participants mounted an anamnestic response, in both vaccine groups, and no differences in post-booster GMTs were evident. This result is consistent with most of the available literature data, which show that, 5–13 years after the completion of the primary course with monovalent recombinant hepatitis B vaccine, more than 86% of subjects with pre-booster anti-HBs levels below 10 mIU/ml responded to the booster vaccination [4,6,8,12,19]. Despite our results are derived by a limited number of observations, they suggest that immune memory persists in children previously vaccinated with hexavalent vaccines.

## Conclusion

Fifteen months after third dose administration, a significant difference in anti-HBs titers was noted in the two vaccine groups considered. Monovalent hepatitis B vaccine administration in 3-year old children induced a proper booster response in both groups, confirming that immunologic memory persists in children with HBsAb titres < 10 mIU/ml.

The administration of anti-poliomyelitis booster was the first chance to evaluate persistence of memory to HBV component after vaccination with hexavalent products, which have been widely used in Italy since 2002. Participant children were evaluated shortly after completion of the primary series.

So, cohorts vaccinated with hexavalent products should be followed to evaluate the trend of anti-HBs titers over time, in order to determine if immunological memory persists during adolescence and adulthood. The administration of a booster dose targeting adolescents, when the risk of infection is likely to increase, should be considered if a decline in immunological memory will be shown.

## Competing interests

The authors declare that they have no competing interests.

## Authors' contributions

CG designed the study, analysed the data, interpreted the results, drafted and edited the manuscript. MLCdA designed the study, interpreted the results, drafted and edited the manuscript. ABe analysed the data. ABa, DM, MM, MO and SZ enrolled and followed participant children and contributed to editing the manuscript. MR, PC and EM conducted the laboratory tests.

## Pre-publication history

The pre-publication history for this paper can be accessed here:


